# A Novel Supramolecular Salt of Hypoxanthine with Maleic Acid as a Potential Weight-Loss Drug

**DOI:** 10.3390/ijms26094266

**Published:** 2025-04-30

**Authors:** Fumin Xue, Xinyue Yuan, Xingyi Li, Shimin Fang, Yan Cheng

**Affiliations:** 1 Shandong Analysis and Test Center, School of Pharmaceutical Sciences, Qilu University of Technology (Shandong Academy of Sciences), Jinan 250014, China; xuefumin@qlu.edu.cn (F.X.); xinyue123452021@163.com (X.Y.); 13846555832@163.com (X.L.); fsm@qlu.edu.cn (S.F.); 2 School of Material Science and Engineering, Shandong Jianzhu University, Jinan 250101, China

**Keywords:** hypoxanthine, maleic acid, solubility, DFT, anti-obesity

## Abstract

Improving the stability of drugs in the solid state, as well as improving their solubility and poor bioavailability, is highly physiologically relevant. In this study, we focused on enhancing the solubility of hypoxanthine (HYP) through salt formation resulting from the preparation of hypoxanthine–maleic acid salt (HYP-MAL). Single crystals were obtained through solvent evaporation methods, and DSC, TGA, PXRD, FT-IR, and ^1^H NMR spectra were used to characterize the samples. The salt system had higher solubility properties than HYP, with an equilibrium solubility in water that was roughly 2.4 times greater than that of HYP, but the salt’s equilibrium solubility increased when the pH shifted from 7.4 to 1.2; additionally, from 0 to 10 min, the powder dissolution rate was 1.8 times that of HYP, resulting in increased bioavailability. The anti-obesity impact of HYP-MAL salt on obese mice was investigated, providing important insights for the development of future weight-loss medications.

## 1. Introduction

HYP, a ubiquitous purine compound found in animals and plants, is a natural purine base produced during purine catabolism [1] that plays a crucial role in regulating physiological functions, energy supply, and metabolic processes in the human body [2,3,4,5], as well as enhancing the taste of cured meat [6]. Furthermore, HYP has demonstrated hepatic accumulation [7], in addition to improved somatosensory-evoked potential recovery and preserved neurofilament 68 kDa protein [8]. Studies have demonstrated its ability to promote lipolysis and reduce body fat mass in mice, making it a common ingredient in health supplements and weight-loss drugs; however, the application of HYP is hindered by its low solubility. One promising strategy through which to increase solubility is the creation of drug cocrystals or salts [9]; numerous studies have confirmed the efficacy of this approach, such as the formation of cocrystals of apixaban with oxalic acid [10], baicalein with isonicotinamide [11], salts of hydrochlorothiazide with nicotinic acid [12] or 2-picolinic acid [12], berberine chloride with fumaric acid [13], ketoconazole with fumaric acid [14], and itraconazole with L-malic acid [15], among others [16,17,18]. Adding cocrystal formers can alter the stacking pattern and improve the stability and solubility of a drug, and converting drug molecules into salt forms is a common strategy through which to increase their solubility, stability, and bioavailability, which can impact various aspects of drug pharmacokinetics [19].

**Scheme 1:**
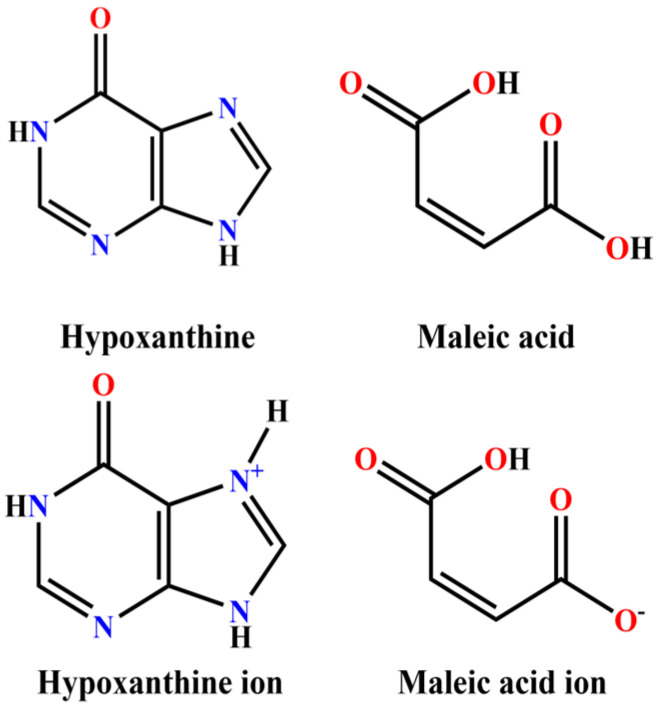
The molecular structures of hypoxanthine, maleic acid, hypoxanthine ions, and maleic acid ions.

There are few studies on the ability of the formation of salts or cocrystals to improve the solubility of HYP. Maleic acid, a widely used salt type [20] with high solubility and good stability [21,22], is commonly employed as a raw material in the production of pharmaceuticals, such as enalapril maleate folic acid tablets and timolol maleate tablets, as well as in cosmetics for moisturizing, antioxidant, and antimicrobial effects. Maleic acid was used as a ligand and HYP as an active pharmaceutical ingredient (API) to create HYP-MAL, a supramolecular ionic salt that was characterized, and its structure examined, in order to address the growing prevalence of obesity worldwide as a result of dietary and lifestyle changes [23,24]; its molecular structures are shown in Scheme 1.

The solubility of HYP-MAL in three different media and the dissolution rate of the powder in phosphate-buffered saline (PBS) were evaluated. Furthermore, a mouse obesity model was established in order to analyze parameters such as body weight, visceral weight, serum lipid levels, liver biochemical indices, fat slices, and the expression of genes related to lipid metabolism. The results of this study provide comprehensive and valuable foundational data for subsequent research into and exploration of the effects of weight loss.

## 2. Results and Discussion

### 2.1. Single-Crystal X-Ray Diffraction (SXRD) Analysis

Gallic acid, 2,4,6-trihydroxybenzoic acid, p-hydroxybenzoic acid, 2,6-dihydroxybenzoic acid, 2,4-dihydroxybenzoic acid, 3,4-dihydroxybenzoic acid, and 1-hydroxy-2-naphthoic acid were all used in our attempts to create single crystals of hypoxanthine. Seven solvent systems, including methanol, methanol/water (*v*/*v* 1:1), ethanol, ethanol/water (*v*/*v* 1:1), acetone/water (*v*/*v* 1:1), methanol/acetone (*v*/*v* 1:1), and methanol/acetone/acetic acid (*v*/*v*/*v* 1:1:0.05), were all used in an attempt to produce various single crystals and salts, but all of them failed. Furthermore, in a single-crystal preparation experiment, HYP to MAL ratios of 2:1, 1:1, 1:2, and 1:5 were investigated, and only the 1:5 ratio produced single crystals.

**Figure 1:**
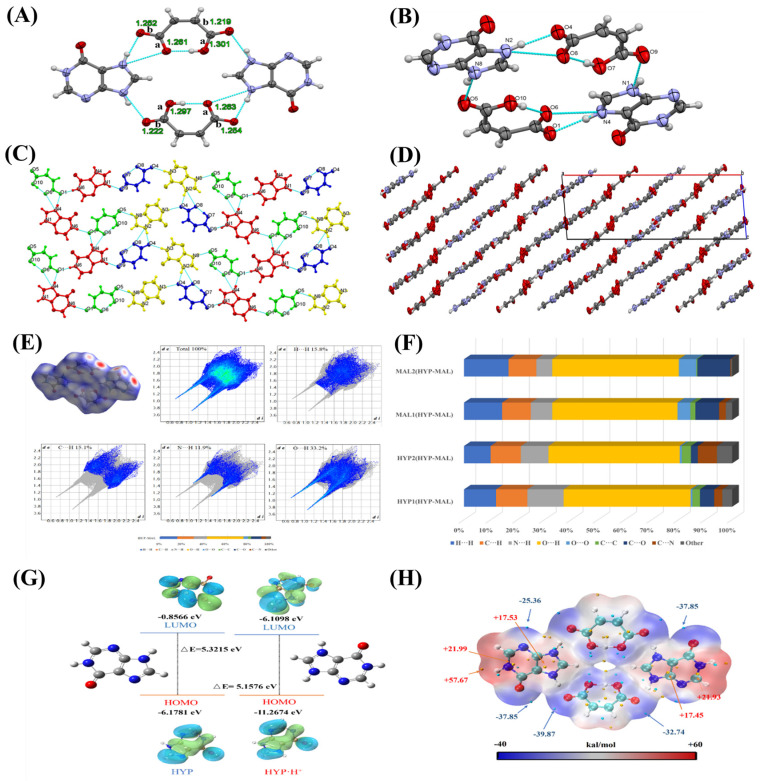
Structure of HYP-MAL: (**A**) the C-O (long) and C-O (short) bond length, including intermolecular (MAL) and intramolecular (HYP-MAL); (**B**) asymmetric unit and hydrogen bond interactions; (**C**) connections of hypoxanthine and maleic acid of HYP-MAL; (**D**) three-dimensional layered crystal structure; (**E**) Hirshfeld surface mapped with d_norm_ and 2D fingerprint plots of HYP-MAL; (**F**) close contact contributions to the Hirshfeld surface areas of HYP-MAL; (**G**) frontier molecular orbital plots, optimized structures, and energy level diagrams of HYP and [HYP]^+^; and (**H**) molecular electrostatic potential surface. (In the molecular representation, oxygen atoms are denoted by the color red, carbon atoms are depicted in gray, nitrogen atoms are illustrated in a pale purple hue, and hydrogen atoms are represented in light blue. Additionally, hydrogen bonds are indicated by green lines.)

The distances of the C-O (long bond) and C-O (short bond) in the carboxylic acids present in the hypoxanthine–maleic acid crystal are detailed in Table S1 and illustrated in Figure 1A. The carboxyl group in maleic acid engages with the imidazole group of HYP, resulting in a C-O (long bond)/C-O (short bond) ratio of 1.007, and induces proton transfer to establish ionic bonds N4^+^−H4···O1^−^ (d, 2.7547 (18) Å, θ, 172°) and N2^+^−H2···O4^−^ (d, 2.7234 (19) Å, θ, 171°). Moreover, maleic acid forms intramolecular hydrogen bonds O10–H10···O6 (d, 2.4192 (17) Å, θ, 177°) and O7−H7···O8 (d, 2.4192 (17) Å, θ, 177°) with C-O (long bond)/C-O (short bond) ratios of 1.067 and 1.061, respectively, without undergoing protonation. On the basis of these data, the hypoxanthine–maleic acid complex was identified as a hypoxanthine–maleic acid salt [25,26]. According to Lange’s *Handbook of Chemistry*, the p*K*a values of hypoxanthine and maleic acid are 5.3 and 3.10, respectively, with a p*K*a difference of 2.20, which is in the ideal zone with regards to the design of salts and cocrystals (−1 ≤ ∆p*K*a ≤ 4) [27].

SXRD revealed that HYP-MAL molecules crystallize in the *P*21/*c* space group, with the asymmetrical unit comprising two hypoxanthine ions and two maleic acid ions (Figure 1B). Two intramolecular hydrogen bonds, O7−H7A···O8 (d, 2.4192 (17) Å, θ, 177°) and O10−H10···O6 (d, 2.4192 (17) Å, θ, 177°), were identified. Additionally, as depicted in Figure 1C, the O−H proton of maleic acid transferred to HYP, resulting in the formation of ionic bonds N2^+^−H2···O4^−^ (d, 2.7234 (19) Å, θ, 171°) and N4^+^−H4···O1^−^ (d, 2.7547 (18) Å, θ, 172°). In the N2-H···O4 salt H bond, the neighboring C-H can form a weak auxiliary bond with O8 through an extra weak H bond C-H···O; the same is true for the C-H···O6 and N4-H···O1 interactions. Furthermore, the interaction of two hypoxanthine ions with two maleate ions via hydrogen bonding—N2−H2···O8 (d, 3.0148 (19) Å, θ, 114°), N1−H1···O9 (d, 2.7370 (20) Å, θ, 158°), N4−H4···O6 (d, 3.0028 (18) Å, θ, 115°), and N8−H8···O5 (d, 2.7050 (20) Å, θ, 161°)—results in the formation of a tetramer. The neighboring tetramers are linked by hydrogen bonding N6−H6···O1 (d, 2.8083 (19) Å, θ, 171°) and N3−H3···O4 (d, 2.8087 (19) Å, θ, 175°) to form the forty-membered ring $R_{8\;6}$(40), which ultimately leads to the formation of a 2D sheet layer; these sheet layers are parallel to each other and form a 3D network structure through π-π stacking (Figure 1D). The crystallographic parameters and the bond lengths and hydrogen bonding angles are presented in Table 1 and Table S2, respectively.

### 2.2. Theoretical Calculation

The visualization of close intermolecular contacts in crystals, analyzed via Hirshfeld surface, provides a method for studying intermolecular interactions. The distance from a point on the Hirshfeld surface to the nearest nucleus inside or outside of the surface can be defined as *d*e and *d*i (*d*_e_, *d*_i_ symmetry), respectively. The normalized contact distance, *d*_norm_, is defined based on *d*_e_, *d*_i_, and the van der Waals radius of the atom. The Hirshfeld surface was utilized to characterize the interaction sites of HYP-MAL, and the Hirshfeld surface of HYP-MAL is depicted in Figure 1E, showing that the surface has been plotted on the *d*_norm_, represented in a blue–white–red color scheme. The red dots represent a *d*_norm_ shorter than the sum of the van der Waals radii, indicating close hydrogen bonding interactions between H and O, displaying the 2D fingerprints and the percentage contributions of contact interactions. The positions of the highlighted blue color and spikes are correlated with the area and strength of the contacts. The 2D fingerprints of HYP-MAL reveal that O···H, H···H, C···H, and N···H contacts are dominant, accounting for 33.2%, 15.8%, 15.1%, and 11.9% of the intermolecular interactions, respectively. Other interactions, such as O···O, account for a negligibly small percentage. The contributions of molecular interactions between each molecule of HYP-MAL can be quantified by analyzing the Hirshfeld surface. As shown in Figure 1F, N−H···O intermolecular hydrogen bonding plays a key role in the construction of the crystal structure. Frontier molecular orbitals are crucial for analyzing a molecule’s electron-donating and accepting capabilities, as well as for understanding its activity and stability during chemical reactions. The optimized geometries and the HOMOs and LUMOs of HYP and [HYP]^+^ are depicted in Figure 1G. The energy level of the HOMO signifies its role as an electron donor, whereas the energy level of the LUMO indicates its ability to accept electrons. The protonation of HYP reduces the HOMO-LUMO energy gap, leading to lower energy absorption by electrons during the transitions from the HOMO to LUMO; consequently, the electron energy distributions become closer, suggesting that [HYP]^+^ is more reactive than HYP, and that HYP is more stable. MEPs are commonly employed to predict and interpret nucleophilic and electrophilic potential sites in molecules. The MEPs of HYP-MAL were analyzed, revealing positive potential regions in red and negative potentials in blue. As illustrated in Figure 1H, the global minimum of HYP-MAL is located on the O atom (−39.87 kcal/mol), and the -OH group loses H to gain electrons, forming [MAL]^−^. The maximum positive potential of HYP-MAL is +57.67 kcal/mol, with the hydrogen atoms on the -NH group acting as hydrogen donors. The N atom on the imidazole ring gains H^+^, with an electrostatic potential of +17.53 kcal/mol distributed over the cation.

### 2.3. Powder X-Ray Diffraction (PXRD) Analysis

**Figure 2:**
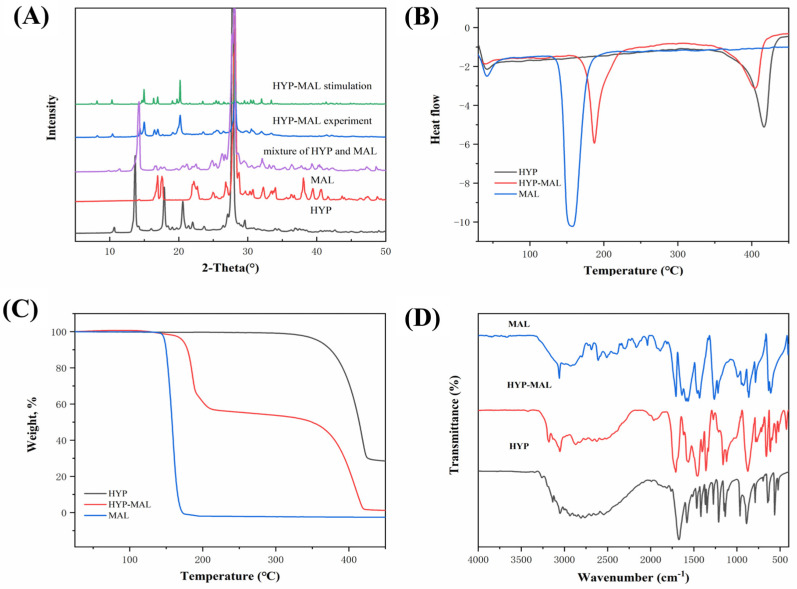
Characterizations of HYP, HYP-MAL, and maleic acid: (**A**) PXRD pattern, (**B**) DSC, (**C**) TGA, and (**D**) FT-IR spectra of HYP, HYP-MAL, and maleic acid.

The production of HYP-MAL was confirmed using PXRD analysis. In contrast to the patterns of HYP and maleic acid, the unique XRD pattern of HYP-MAL, as displayed in Figure 2A, validates the creation of a novel crystal phase. For HYP, the distinctive diffraction peaks, at 2θ, of 10.6°, 14.2°, 17.9°, 18.4°, 19.1°, 23.7°, 26.4°, and 27.1°, vanished, as did the corresponding peaks for maleic acid at 16.9°, 17.6°, 25.4°, 26.8°, 28.2°, and 28.7°. In the HYP-MAL pattern, new characteristic peaks emerged at 7.7°, 9.9°, 13.4°, 13.9°, 16.5°, 17.2°, 18.7°, and 23.1°, which shows that the original samples have been completely consumed and that new crystalline phases are present. Evidence for the creation of a new crystalline phase was also provided through a comparison with the physical mixture profile, which showed notable variations in the quantity and relative intensity of distinctive diffraction peaks. Significant variations in the quantity and relative intensity of distinctive diffraction peaks were also seen when compared to the physical mixing profile, supporting the creation of a new crystalline phase. Further evidence of HYP-MAL’s high purity comes from the overlap between its pattern and the simulated pattern from the SXRD study.

### 2.4. Thermal Analysis

HYP-MAL’s thermal characteristics were examined using thermogravimetric analysis (TGA) and differential scanning calorimetry (DSC). Endothermic peaks for HYP and maleic acid were found at 416.83 °C and 155.50 °C, respectively, as shown in Figure 2B,C. On the other hand, HYP-MAL showed endothermic peaks, which are suggestive of a melting process, at 186.83 °C and 407.83 °C, demonstrating that the formation of salt improves the stability of the HYP-MAL molecule. The unique melting point of HYP-MAL, which is different from the melting points of its constituent parts, indicates that a new phase may have formed as a result of their interaction. TGA analysis showed that the melting of HYP-MAL was immediately followed by degradation (Figure 2C), with 71.47% of HYP’s mass lost by the time melting occurred; in comparison, MAL completely lost its weight around 190 °C after melting. HYP-MAL displayed two separate weight-loss phases, with the first phase resulting in a 45.67% loss, which aligns with the loss of maleic acid (theoretical weight loss of 46.04%), occurring at a temperature lower than that of pure maleic acid’s decomposition. The second weight-loss phase, of 53.30%, corresponded to the loss of HYP molecules.

### 2.5. FT-IR and ^1^H NMR Spectroscopy

The red shift in intensity is linked to the strength of hydrogen bond formation, with greater intensity correlating with a larger red shift. The presence of hydrogen bonding also led to the broadening of the vibrational peaks. The infrared spectra of HYP, maleic acid, and HYP-MAL are depicted in Figure 2D. The characteristic peak of HYP at 1709 cm^−1^ corresponds to the stretching vibration of C=O, while the N-H stretching vibrations appear at 3177 cm^−1^ and 3052 cm^−1^. Maleic acid exhibited a characteristic peak at 1710 cm^−1^ for the C=O stretching vibration, as well as a broader peak at 3060 cm^−1^ for O-H. Upon the formation of HYP-MAL, the N-H peak of HYP shifted from 3177.5 cm^−1^ to 3135.87 cm^−1^, indicating a decrease in electron cloud density and a stronger hydrogen bonding force. Additionally, the C=O peak of maleic acid shifted from 1710 cm^−1^ to 1671.91 cm^−1^, suggesting its involvement in hydrogen bonding and a change in the mode of hydrogen bonding action, resulting in broadening of the vibrational peaks.

The ^1^H NMR spectra and chemical shifts of HYP-MAL, HYP, and maleic acid are depicted in Figures S1–S3; according to the integration results, the stoichiometric ratio of HYP to maleic acid was 1:1.

### 2.6. Equilibrium Solubility

**Figure 3:**
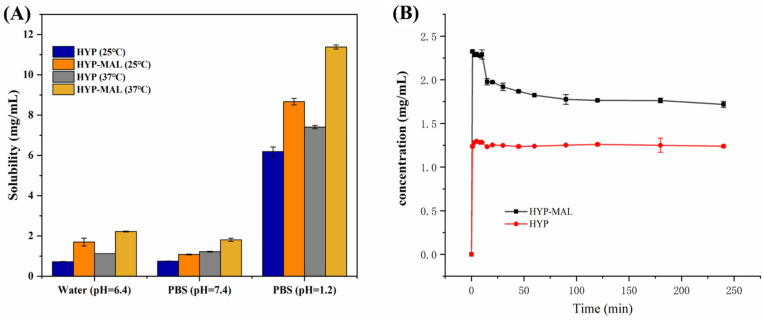
(**A**) Equilibrium solubilities of HYP and HYP-MAL at different pH values and temperatures (n = 3). (**B**) Powder dissolution profiles of HYP and HYP-MAL in PBS (37 °C).

The relationship between solubility and bioavailability is well established, with increased solubility often leading to increased bioavailability. In this study, the solubilities and equilibrium solubilities of HYP and HYP-MAL were investigated using high-performance liquid chromatography (HPLC) (Figure 3A). The solid powder samples were introduced into water and PBS at various temperatures and pH values and subsequently stirred for 24 h [28]. The findings revealed that the solubility of HYP in HYP-MAL was notably greater than that of HYP alone, in both water and PBS, at room temperature (25 °C) and 37 °C; furthermore, the solubility of the salt was found to be twice that of HYP in water at pH = 6.4.

### 2.7. Powder Dissolution Experiments

The dissolution rate of HYP in HYP-MAL was also observed to be higher than that of HYP alone, indicating a “spring-parachute” effect (Figure 3B). Although the dissolution rate of HYP in HYP-MAL decreased slightly over time, it remained consistently higher than that of HYP alone, a phenomenon that can be attributed to solvent–solute interactions, which disrupt the intermolecular bonds of the salt and alter the pH of the solution, thereby increasing the potential of the solute to form hydrogen bonds with water molecules, consequently increasing its solubility; these findings underscore the potential of salt formation in enhancing solubility-limited bioavailability.

### 2.8. Anti-Obesity Effect of HYP-MAL in Nutritionally Obese Mice

**Figure 4:**
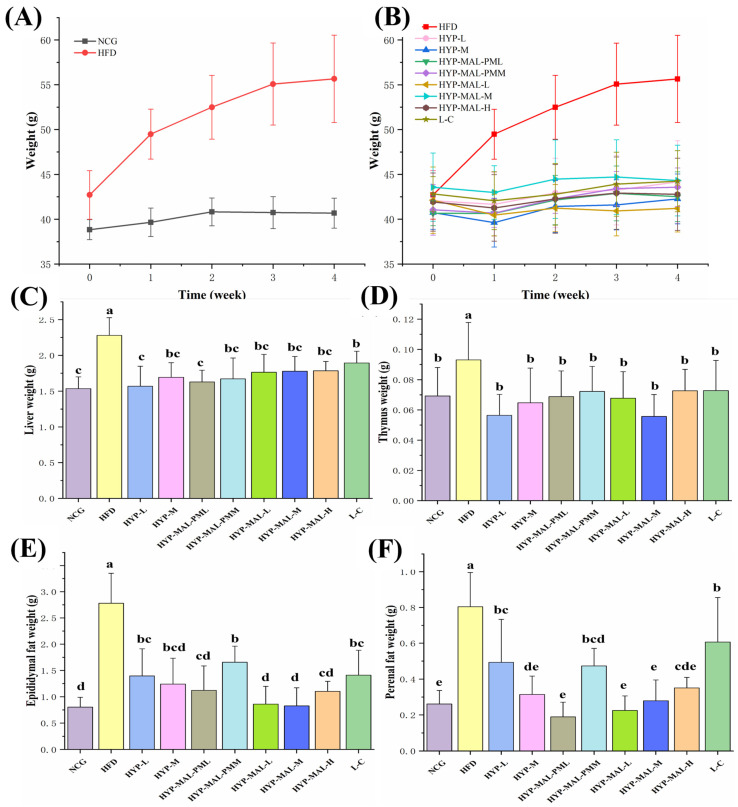
Body weights and changes in the mice during the dosing period (n ≥ 6): (**A**) HFD group and NCG, and (**B**) HFD, HYP-MAL-PML, HYP-MAL-PMM, HYP-MAL-L, HYP-MAL-M, HYP-MAL-H, and L-C. Effects of HYP-MAL on the internal organs of mice (n ≥ 6): (**C**) liver, (**D**) thymus, (**E**) epididymal fat, and (**F**) perirenal fat. ^abc^ The means not sharing a common letter are significantly different between groups (*p* < 0.05). NCG: normal control group; HFD: high-fat diet group; HYP-MAL-L: low-dose HYP-MAL salt group; HYP-MAL-M: medium-dose HYP-MAL salt group; HYP-MAL-H: high-dose HYP-MAL salt group; L-C: *L* (-)-carnitine.

As depicted in Figure 4A, after four weeks of high-fat chow feeding (week 0), the body weights of the mice in the treatment group exceeded those of the normal control group (NCG) mice, indicating successful modeling (Table S3). Throughout the gavage process, the body weights of the mice in the high-fat diet (HFD) group consistently surpassed those of the mice in the NCG, with the disparity in body weight between the two groups progressively widening over time. Upon drug intervention, the body weights of the mice in each dosage group were notably lower than those in the HFD group from the onset of drug administration, indicating a significant difference. The drug substantially inhibited the increase in body weight of the mice; however, owing to the potential destruction of the salt structure in the strongly acidic gastric environment and poor absorption, no significant difference was observed between the dosing groups. Figure 4B and Table S4 illustrate the alterations in the body weights of the mice during the administration period, as well as the differences before and after the administration of HYP-MAL. HYP-MAL-L, HYP-MAL-M, and HYP-MAL-H represent the low, medium, and high dose groups, respectively; the weight gain result in the low dose group is a negative number, while the weight gain results in the medium and high dose groups of HYP-MAL were lower than those in the other administration groups. Compared with the other groups, HYP-MAL-L resulted in negative body weight gain, whereas HYP-MAL-M and HYP-MAL-H exhibited lower body weight gain, indicating superior weight loss. The subsequent HYP-MAL groups, denoted HYP-MAL-L, HYP-MAL-M, and HYP-MAL-H, further displayed favorable weight loss. The HYP-MAL group was subjected to additional investigations and discussion in subsequent experiments.

Changes in the liver, thymus, and fat mass in mice serve as important indicators of obesity severity. Visceral fat-induced obesity is linked to hypertension, dyslipidemia, and diabetes mellitus, as depicted in Figure 4C–F. The study findings revealed significant increases in liver, thymus, epididymal fat, and perirenal fat weights in HFD-fed mice, approximately 1.5, 1.4, 3.5, and 3.1 times higher than those in the NCG-fed mice and closely associated with HFD consumption; conversely, the L-C and intervention groups presented significant reductions in liver, thymus, epididymal fat, and perirenal fat weights. Notably, the liver weights in the HYP-MAL groups did not significantly differ from those in the NCG, but the effect was significant compared with that in the HFD group; furthermore, the epididymal fat weights in the HYP-MAL group mice were 69%, 70%, and 60% lower than those in the HFD group and did not significantly differ from those in the NCG; similarly, the effect of HYP-MAL on perirenal fat weights did not significantly differ from that of the NCG but were significantly lower than that of the HFD group, representing 28%, 35%, and 44% of the HFD weight, respectively.

**Figure 5:**
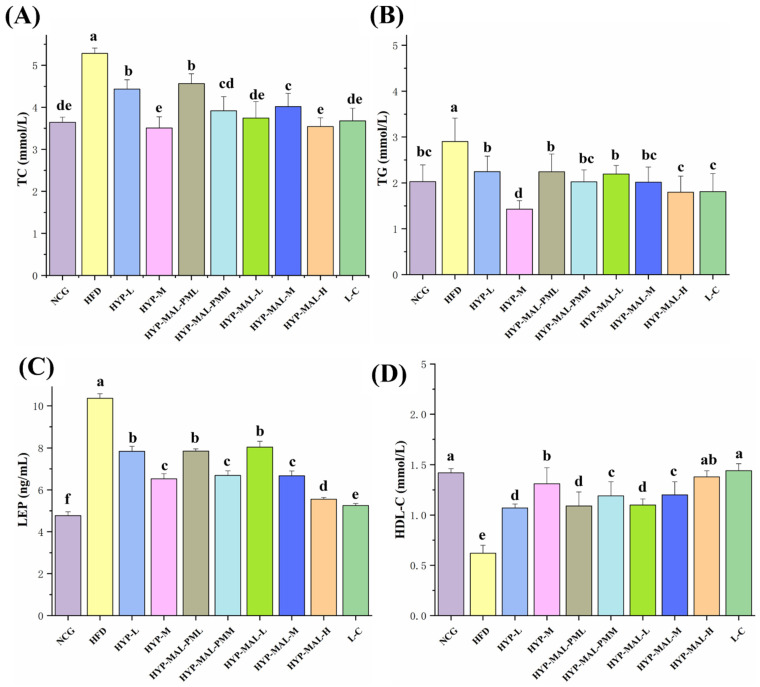
Effects of HYP-MAL on the serum levels of obese mice (n ≥ 6): (**A**) TG, (**B**) TC, (**C**) LEP, and (**D**) HDL-C. ^abcdef^ The means not sharing a common letter are significantly different between groups (*p* < 0.05). NCG: normal control group; HFD: high-fat diet group; HYP-MAL-L: low-dose HYP-MAL salt group; HYP-MAL-M: medium-dose HYP-MAL salt group; HYP-MAL-H: high-dose HYP-MAL salt group; L-C: *L* (-)-carnitine.

High-fat diets have been associated with lipid metabolism disorders, particularly dyslipidemia [29]. After four weeks of modeling, the HFD-fed mice were randomly selected, and the serum concentrations of total cholesterol (TC) and triglycerides (TG) were found to be significantly greater than those in the NCG-fed mice, indicating successful model establishment (Table S5). As depicted in Figure 5A–D, the high-fat diet led to elevated serum levels of TC and TG, as well as decreased levels of HDL-C in the mice. However, following intervention with HYP-MAL, the serum concentrations of TC and TG in the treatment groups were significantly lower than those in the HFD group, suggesting the ability of HYP-MAL to mitigate high-fat diet-induced dyslipidemia in mice; furthermore, high-fat diets have been shown to elevate lipocalin levels and increase leptin levels [30]. ELISA was utilized to investigate the impact of the drug on leptin (LEP) levels in the serum of obese mice fed a high-fat diet. Compared with that in the NCG, the serum level of LEP in the HFD group was significantly elevated, nearly 2.5 times greater, whereas the level of LEP in the HYP-MAL-H group closely resembled those in the L-C group and the NCG. Additionally, the HDL-C level in the HFD-fed mice was significantly lower than that in the NCG-fed mice but increased following drug intervention, with no significant differences observed between the HYP-MAL-H and L-C groups and the NCG.

**Figure 6:**
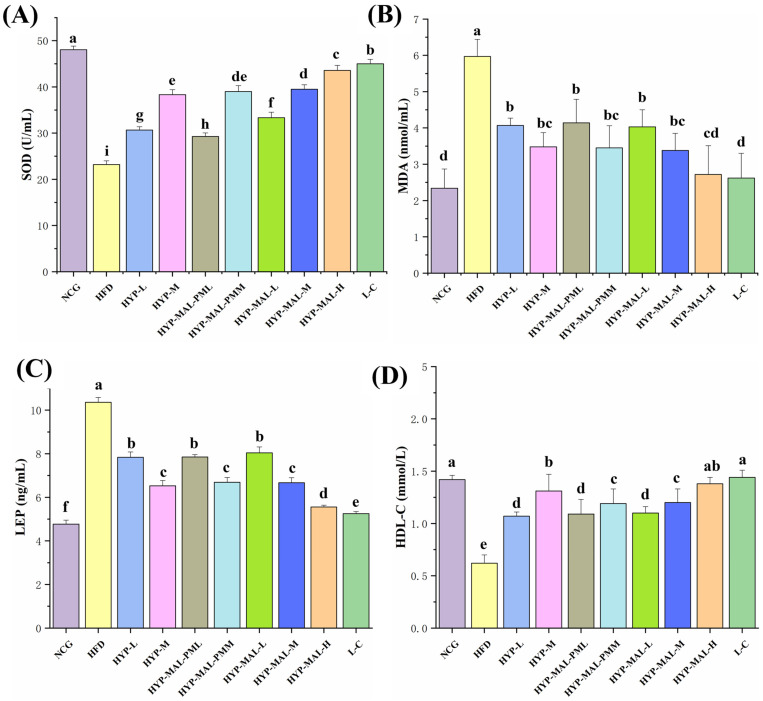
Gene expression levels of (**A**) LEP and (**B**) ADP in mice. Effects of HYP-MAL on liver tissue levels in mice: (**C**) SOD and (**D**) MDA. ^abcdef^ The means not sharing a common letter are significantly different between groups (*p* < 0.05). NCG: normal control group; HFD: high-fat diet group; HYP-MAL-L: low-dose HYP-MAL salt group; HYP-MAL-M: medium-dose HYP-MAL salt group; HYP-MAL-H: high-dose HYP-MAL salt group; L-C: *L* (-)-carnitine.

Superoxide dismutase (SOD) is an in vivo free radical-scavenging enzyme that indirectly reflects the antioxidant capacity of an organism. Malondialdehyde (MDA) is a cytotoxic degradation product of in vivo peroxidation reactions that causes damage to the cell membrane structure. The enzyme activities of SOD and MDA, which can be used to assess liver damage in mice, are presented in Figure 6A,B as the results of each group after four weeks of drug intervention. Compared with that in the NCG, SOD enzyme activity was significantly lower, and MDA enzyme activity was significantly greater, in the livers of HFD-fed mice (*p* < 0.05), indicating oxidative damage to the liver. In the HYP-MAL group, SOD enzyme activity was elevated, and liver MDA enzyme activity was decreased, compared with the HFD group, indicating a protective effect against oxidative damage. In the HYP-MAL-H group, SOD enzyme activity was similar to that in the NCG and L-C groups, and there was no significant difference in MDA enzyme activity compared with that in the NCG and L-C groups, demonstrating that HYP-MAL-H was the most effective intervention.

To evaluate the rationality of primer preparation, the dissolution curves of the three primer pairs were examined. As depicted in Figure S4, the dissolution curves exhibited a distinct single peak exceeding 80, indicating successful preparation of the experimental primers and the reliability of the obtained data for subsequent analysis. The results of the qPCR analysis of LEP mRNA and ADP mRNA gene expression are presented in Figure 6C,D. In comparison to the NCG, LEP mRNA expression was significantly elevated, whereas ADP mRNA expression was significantly reduced in the HFD group. Following intervention with each drug, the alterations in gene expression were mitigated and normalized in a dose-dependent manner, with HYP-MAL-H and L-C demonstrating superior efficacy. According to the determination of various indicators, the medium (HYP-MAL-M) and low dose groups (HYP-MAL-L) demonstrated favorable anti-obesity effects. Gu et al. discovered through measurements of aspartate aminotransferase and alanine aminotransferase in liver homogenate that HYP may induce potential toxicity and damage to the liver [31].

**Figure 7:**
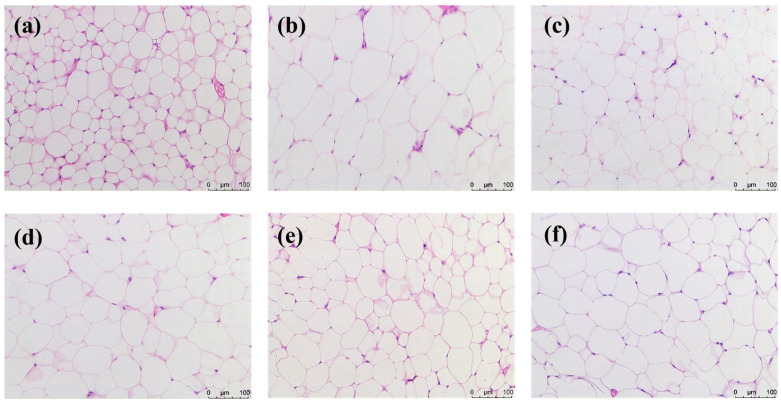
Histological images of mouse adipose tissues (×200): (**a**) NCG, (**b**) HFD, (**c**) HYP-MAL-L, (**d**) HYP-MAL-M, (**e**) HYP-MAL-H, and (**f**) L-C. NCG: normal control group; HFD: high-fat diet group; HYP-MAL-L: low-dose HYP-MAL salt group; HYP-MAL-M: medium-dose HYP-MAL salt group; HYP-MAL-H: high-dose HYP-MAL salt group; L-C: *L* (-)-carnitine.

After drug administration, microscopic observation of abdominal adipose tissue sections from each group revealed distinct differences. As depicted in Figure 7, the adipose sections of NCG mice exhibited uniform round or polygonal adipocytes with blue or flat round cell nuclei; in contrast, adipose tissue sections from HFD-fed mice were larger in size and irregular in shape and presented elevated intracellular fat content, unclear cell membranes, and easily fusible cell edges. Following drug intervention, a significant reduction in adipose tissue cell size was observed, indicating potential recovery. Notably, HYP-MAL-H drug intervention resulted in a notable alteration in adipocyte morphology, with a marked decrease in the number of large fat droplets and improved delineation and regularity of adipocytes in terms of volume size and morphology, findings which suggest that the drug inhibits the adipose hypertrophy and fat droplet accumulation induced with dietary sources, thereby improving adipocyte growth and expansion while reducing fat accumulation and alleviating obesity symptoms.

## 3. Experimental Procedures

### 3.1. Materials

HYP was procured from Shanghai Aladdin Biochemical Technology Co., Ltd. (Shanghai, China), whereas maleic acid was sourced from Shanghai Macklin Biochemical Co., Ltd., Shanghai, China. The solvents utilized in this study, namely methanol and acetone, were obtained from Sinopharm Chemical Reagent Co., Ltd., Shanghai, China. Deionized water was produced in the laboratory via an Arium Mini plus system from Germany. For detailed information on the chemicals used in this study, please refer to Table S6 in the Supporting Information. The hyperlipidemia model feed developed by Beijing Keao Xieli Feed Co., Ltd. (Tianjin, China), consists of basic feed supplemented with 15% lard, 20% sucrose, 1.2% cholesterol, 0.2% sodium cholate, and other components, detailed in Table S7.

### 3.2. Preparation Methods

The HYP-MAL composite was prepared via the liquid-assisted grinding method [32] and the solution evaporation method [33]. For the liquid-assisted grinding method, HYP (34.03 mg, 0.25 mmol) and maleic acid (29.02 mg, 0.25 mmol) were mixed with 30.0 µL methanol in a 5 mL ball mill (Retsch, 1.4112) and ground for 5 min with two 7 mm stainless steel balls at a frequency of 26 Hz. For the solution evaporation method, HYP (13.61 mg, 0.10 mmol) and maleic acid (58.04 mg, 0.50 mmol) were dissolved in a solvent mixture containing 10.0 mL of methanol and 10.0 mL of acetone, heated, stirred for 4 h, and filtered, after which a small amount of water was added. The resulting filtrate was left at room temperature for approximately 20–30 days to obtain high-quality crystals.

### 3.3. General Characterization

PXRD patterns were obtained via a MiniFlex 600 powder diffractometer (Rigaku Corporation, Tokyo, Japan), and single-crystal structures were determined with a Rigaku Oxford Diffraction XtaLAB Synergy-S diffractometer. Fourier transform infrared (FT-IR) spectra were recorded using a Vertex 70 FT-IR spectrometer (Germany), and nuclear magnetic resonance (NMR) spectra were acquired on an Avance NEO 400 MHz spectrometer (Bruker, Berlin, Germany) with (methyl sulfoxide)-*d*_6_ as the solvent. The decomposition (T_d_) and melting temperatures (T_m_) were determined using a thermogravimetric analysis/differential scanning calorimetry (TGA/DSC) 3+ instrument (METTLER TOLEDO, Greifensee, Switzerland).

### 3.4. HPLC Analysis

An Agilent 1260 HPLC system, equipped with a G7114A 1260 VWD detector, a G7129A 1260 Vial sampler, and a G7111B 1260 Quat Pump, was obtained from Agilent Technologies Co., Ltd. (model 1260, Santa Clara, CA, USA). Additionally, an InertSustain C18 column (5 μm, 4.6 × 250 mm) was purchased from Shimadzu Corporation (Kyoto, Japan). The mobile phase was prepared by measuring 1.00 ± 0.02 mL of phosphoric acid in a 1000.0 mL volumetric flask, adding 100.0 mL of methanol (HPLC grade) at a fixed volume to scale with deionized water, and sonicating to remove air bubbles. The injection volume was 2.0 μL, and the mobile phase flow rate was 0.6 mL/min. The separation column was maintained at 30 °C, and UV detection was set at 250 nm. A standard graph for the HYP component was constructed by plotting the concentration versus area, resulting in the equation *Y* = 7457.2*X* − 4.1093, with an *R*^2^ value of 0.9999, where *Y* represents the area of the peak, *X* represents the amount of HYP in micrograms, and *R* represents the relative standard deviation.

### 3.5. Equilibrium Solubility and Dissolution Experiments

For equilibrium solubility and dissolution tests, HYP and HYP-MAL were each milled and sieved separately using standard mesh sieves, with each experiment carried out in triplicate. Excess HYP was stirred in 10.0 mL of solvent at 25/37 °C, with pH values of 1.2 PBS, 6.4 water, and 7.4 PBS. The process using HYP-MAL was the same. For HPLC analysis, the resultant slurry was filtered and suitably diluted. Tests of powder dissolution were performed in intestinal fluid simulations (PBS, pH = 7.4). At 37 °C, HYP and HYP-MAL were shaken at 100 rpm in 40.0 mL of PBS. Samples were taken at 1, 3, 5, 8, 10, 15, 20, 30, 45, 60, 90, 120, 180, and 240 min. At predetermined intervals, 1.00 mL of the solution was taken out and filtered through 0.22 μm filters, after which 1.00 mL of new dissolution medium was immediately added. HPLC was used to measure the concentration once the filtrate had been suitably diluted.

### 3.6. Density Functional Theory (DFT) Study

Hirshfeld surface analysis [34] was conducted to determine the relative contributions of various intermolecular contacts in HYP-MAL. Crystal View 9.2.5 was used to generate Hirshfeld surface maps and 2D fingerprint plots. Additionally, extensive DFT calculations were employed to comprehend and predict intermolecular interaction forces. The B3LYP functional was utilized for all calculations, and the 6-31G (d) basis set was employed for hydrogen atom geometric optimizations and single energy calculations; the Gaussian 09 package was used for all calculations. Furthermore, the molecular electrostatic potential surface [35] (MEPs) and molecular orbitals (highest occupied molecular orbitals, HOMO; lowest unoccupied molecular orbitals, LUMO) were calculated using Multiwfn software [36,37] and visualized with VMD software [38].

### 3.7. Animal Model and Subject Details

#### 3.7.1. Ethics Statement

The present study received approval from the ethics committee of Shandong University of Traditional Chinese Medicine (No. SUTCM 2015-01-03).

#### 3.7.2. Animals

In this study, we used 80 three-week-old ICR male mice, with body weights ranging from 18 to 22 g (License No., SCXK 2021-0006), obtained from Beijing Vital River Laboratory Animal Technology Co., Ltd., Beijing, China. All animals were housed in the Animal Experimentation Center of Shandong University of Traditional Chinese Medicine (SUTCM).

#### 3.7.3. Culture of Normal and High-Fat Mice

Mice were cultured for one week at 55 ± 1% relative humidity, 23 ± 1 °C temperature, and a 12 h light–dark cycle. Some mice were then randomly allocated to the normal control group (NCG) and fed regular chow for the duration of the trial. The remaining mice were fed a mixture of high-fat and regular chow for one week before being fed only high-fat chow. After four weeks, mice in the high-fat diet (HFD) group were randomly selected to have their total cholesterol (TC), total triglyceride (TG), and high-density lipoprotein cholesterol (HDL-C) levels measured. Mice that met the experimental conditions were included in the study.

#### 3.7.4. Groups and Dosage

The groups and their dosages are shown in Table S8.

Each group contained a minimum of six mice. HFD and NCG conditions remained consistent throughout the experiment, with the exception of feed and gavage medication administration. The NCG received an equivalent volume of saline through gavage. Weekly body weight measurements were performed, and the food intake of the mice in each group was recorded. The anesthetic used was 20% ethyl carbamate mixed with physiological saline, administered intraperitoneally at a rate of 0.1 mL/10 g. Additionally, the mice’s body length was assessed before and after medication delivery.

#### 3.7.5. Sample Collection

Following four weeks of modeling, the mice’s orbital sinuses were used to draw blood samples for TG, TC, and HDL-C serum level examination. After receiving the medication via gavage for four weeks in a row, the mice were given limited access to water and fasted for 14–16 h at the conclusion of the fourth week. Blood was drawn from the orbital sinus after this fasting interval and left to clot for an hour at room temperature. To extract the serum, the samples were then centrifuged for 15 min at 4 °C and 3000 rpm. The organs required for the experiment were then dissected and weighed after the mice were euthanized. The remaining tissues were kept in an ultralow-temperature refrigerator at −80 °C, while the epididymal fat was fixed with a specific fixative for hematoxylin and eosin (H&E) staining.

#### 3.7.6. Blood Lipid Testing

Blood lipid testing was performed via an enzyme labeling apparatus and corresponding biochemical kits. The GPO-PAP enzymatic method was employed to measure TG levels, the COD-PAP method was utilized for TC detection, and the direct method was employed for HDL-C content determination.

#### 3.7.7. Measurement of Serum Leptin (LEP) and Liver Adiponectin (ADP) Levels

The LEP content in the serum and the ADP content in the liver of each group of mice were measured using the enzyme-linked immunosorbent assay (ELISA) following the provided instructions.

#### 3.7.8. Determination of Liver Malondialdehyde (MDA) Content and Superoxide Dismutase (SOD) Activity

Liver tissue was accurately weighed, homogenized in precooled PBS, and then centrifuged. The content of MDA and the activity of SOD were determined via specific assay kits according to the manufacturer’s instructions.

#### 3.7.9. Histological Staining of Adipose Tissue

Epididymal adipose tissue was fixed using a specialized fixative for adipose tissue, followed by sequential steps of washing, dehydration, and embedding in wax. The tissue was then sectioned and stained with H&E to visualize the histological features.

#### 3.7.10. RNA Extraction and Gene Expression Analysis

RNA was extracted from liver tissues, and relative gene expression was analyzed. The sequences of primers used for gene detection are provided in Table S9. Approximately 30.0 mg of liver tissue from experimental animals was homogenized, lysed, and purified at low temperature via a high-throughput tissue homogenizer to obtain total RNA. The RNA concentration was measured using an ultra-micro spectrophotometer. Subsequently, 20.0 μL of RNA was reverse transcribed into the first strand of cDNA. The target gene was amplified through quantitative polymerase chain reaction (Q-PCR) under specific conditions. The results of the Q-PCR were analyzed using the 2-ΔΔCt method, where a value > 1 indicates increased gene expression compared with the control group, and a value < 1 indicates decreased gene expression.

#### 3.7.11. Statistical Analysis

The obtained results were analyzed via one-way analysis of variance (ANOVA) in SPSS 16.0 statistical software, and the difference between means was determined using Duncan’s multiple range test. The experimental data are expressed as means ($\bar{x}$) ± standard deviations (s). Statistical significance was considered at *p* < 0.05.

## 4. Conclusions

In this study, HYP-MAL was effectively synthesized using liquid-assisted milling and solvent evaporation procedures with a molar ratio of 1:1; a single-crystal structure was solved, revealing an asymmetric unit made up of two hypoxanthine ions and two maleic acid ions, held together by hydrogen bonding and ionic interactions. A detailed analytical characterization of HYP-MAL was carried out, including PXRD analysis, which confirmed the salt’s unique crystalline phase, high purity, and agreement with the single-crystal simulation pattern. The FT-IR revealed variations in covalent bond lengths and energy due to hydrogen bonding, as well as changes in hydrogen bonding modes caused by salt production. A study of the increase in HYP-MAL solubility using powder dissolution experiments and equilibrium solubility determination revealed improved solubility and the potential for greater bioavailability. The salt’s equilibrium solubility was at its maximum at pH 1.2, and theoretical molecular computations indicated the probability of salt production. Furthermore, an anti-obesity study employing an obesity mouse model found that HYP-MAL had significant anti-obesity benefits, with HYP-MAL-H having the greatest effect. Overall, the results of this study provide thorough data that support HYP research and provide valuable foundational data for further exploration of weight-loss and hypertension treatments.

## Figures and Tables

**Table 1:** Crystal data and structure refinement parameters for HYP-MAL.

Parameters	HYP-MAL
Moiety formula	C_5_H_4_N_4_O, C_4_H_3_O_4_
Sum formula	C_9_H_7_N_4_O_5_
MW	251.19
Crystal system	Monoclinic
Space group	*P*21/*c*
Hall group	$\bar{P}$2*ybc*
a (Å)	24.3661 (5)
b (Å)	12.2508 (3)
c (Å)	7.0398 (2)
α (deg)	90
β (deg)	95.862 (2)
γ (deg)	90
Volume	2090.42 (9)
Z	8
Data completeness	0.989
R (reflections)	0.0395 (2974)
wR_2_ (reflections)	0.1192 (3702)
S	1.049
Npar	327

## Data Availability

Detailed Information of the Chemicals (PDF) and CCDC 2281943 contain the supplementary crystallographic data for this paper, which can be obtained free of charge via https://www.ccdc.cam.ac.uk/structures/ (accessed on 26 April 2025).

## Supplementary Materials

The following supporting information can be downloaded at: https://www.mdpi.com/article/10.3390/ijms26094266/s1, Figure S1: *I*H NMR·spectra of HYP-MAL; Figure S2: *I*H NMR·spectra of HYP; Figure S3:*I*H NMR·spectra of HYP; Figure S4:Amplification and dissolution curves of (a) LEP and (b) ADP; Table S1: C-O (long) and C-O (short) bond lengths and ratios of HYP-MAL; Table S2: Hydrogen-bond geometry statistics for HYP-MAL; Table S3: Changes in the body weights of mice fed a high fat diet for 4 weeks ($\bar{x}$ ± s); Table S4: Changes in the body weights of the mice before and after drug administration (*n* ≥ 6) ($\bar{x}$ ± s); Table S5: Serum TG, TC and HDL-C concentrations of the mice before administration; Table S6: Detailed information of experimental materials; Table S7: Formula for high fat feed; Table S8: Groups and dosages; Table S9: Detection of primer sequence information
