# Supplementary material for: A Novel Supramolecular Salt of Hypoxanthine with Maleic Acid as a Potential Weight-Loss Drug
Source: Int J Mol Sci. 2025 Apr 30;26(9):4266. doi: 10.3390/ijms26094266 (PMC12071735; doi:10.3390/ijms26094266)
Supplement: Supplementary file 1 [file ijms-26-04266-s001.zip › ijms-3554829-supplementary.pdf]

Supporting Information

Contents

1. Supplementary Tables

2. Supplementary Figures

Table S1 C-O (long) and C-O (short) bond lengths and ratios of HYP-MAL

	C-O (a: Long bond)	C-O (b: Short bond)	Specific value (a/b)
Maleic acid 1	1.261	1.252	1.007
Maleic acid molecule 1	1.301	1.219	1.067
Maleic acid 2	1.263	1.254	1.007
Maleic acid molecule 2	1.297	1.222	1.061

Table S2 Hydrogen-bond geometry statistics for HYP-MAL.

D-H···A	D-H [Å]	H···A [Å]	D···A [Å]	D-H···A [Å]
N1−H1···O9	0.86	1.92	2.7370 (20)	158
N2−H2···O4	0.86	1.87	2.7234 (19)	171
N2−H2···O8	0.86	2.56	3.0148 (19)	114
N3−H3···O4[1#]	0.86	1.95	2.8087 (19)	175
N4−H4···O1	0.86	1.90	2.7547 (18)	172
N4−H4···O6	0.86	2.54	3.0028 (18)	115
N6−H6···O1[2#]	0.86	1.96	2.8083 (19)	171
O7−H7A···O8	0.82	1.60	2.4192 (17)	177

N8−H8···O5	0.86	1.88	2.7050 (20)	161
O10−H10···O6	0.82	1.60	2.4192 (17)	177

Symmetry codes: 1# -x, -1/2+y, 1/2-z; 2# 1-x, 1/2+y, 5/2-z

Strong H bonds were listed

Table S3 Changes in the body weights of mice fed a high fat diet for 4 weeks ($\bar{x} \pm s$)

Group	Weight (g)
Normal group	38.84 ± 1.11
High fat group	42.12 ± 0.90

Table S4 Changes in the body weights of the mice before and after drug administration ($n \geq 6$) ($\bar{x} \pm s$)

Group	Before administration (g)	After administration (g)	Differential value (g)
NCG	38.84 ± 1.11	40.68 ± 1.67	1.84
HFD	42.71 ± 2.71	55.66 ± 4.87	12.95
HYP-MAL-L	42.13 ± 3.71	41.20 ± 2.61	-0.93
HYP-MAL-M	43.58 ± 3.80	44.32 ± 3.95	0.74
HYP-MAL-H	41.91 ± 3.24	42.77 ± 4.03	0.86
L-C	42.82 ± 1.93	44.24 ± 3.41	1.42

Table S5 Serum TG, TC and HDL-C concentrations of the mice before administration

Group	TG (mmol/L)	TC (mmol/L)	HDL-C (mmol/L)
NCG	1.07 ± 0.19	5.12 ± 0.53	3.08 ± 0.33
HFD	1.87 ± 0.37	7.67 ± 1.63	3.70 ± 0.89

Table S6 Detailed information of experimental materials

Materials	CAS/CAT	Molar mass ($g·mol^{-1}$)	Source	Purity (%)	Analysis method
Hypoxanthine	68-94-0	136.11	Shanghai Macklin Biochemical Co., Ltd.	≥ 99	HPLC [a]
Maleic acid	110-16-7	116.07	Shanghai Macklin Biochemical Co., Ltd.	≥ 99	HPLC [a]
PBS	MA0008	/	Dalian Meilunbio Co., Ltd.	/	/
L(-)-Carnitine	C15832528	161.20	Shanghai Macklin Biochemical Co., Ltd.	≥ 98	HPLC [a]
Phosphoric acid	7664-38-2	97.99	Sinopharm Chemical Reagent Co., Ltd.	≥ 85.0	/
Methanol	67-56-1	32.04	Sinopharm Chemical Reagent Co., Ltd.	≥ 99.7	GC [b]
Ethanol	20220708	46.07	Sinopharm Chemical Reagent Co., Ltd.	≥ 99.5	GC [b]
Acetone	67-64-1	58.08	Sinopharm Chemical Reagent Co., Ltd.	≥ 99.7	GC [b]
Saline	B22060401B	/	Shandong Kelun Pharmaceutical Co., Ltd.	/	/
Dimethylbenzene	20240122	106.16	Fuyu Chemical Co., Ltd.	≥ 99.0	GC [b]
Hydrochloric acid	20240117	/	Fine Chemical Plant in Laiyang Economic and Technological Development Zone	36-38%	/
Neutral balsam	20240103	/	Beijing Solarbio Science & Technology Co., Ltd.	/	/
Acetic acid	210712497	60.05	Nanjing Reagent	≥ 99.0	GC [b]
Ethyl carbamate	51-79-6	89.09	Beijing Solarbio Science & Technology Co., Ltd.	≥ 98	GC [b]
Special fixative solution for fat	CR2210071	/	Wuhan Servicebio Technology Co., Ltd.	/	/
Total cholesterol (T-CHO) assay kit	20231113 20240124	/	Nanjing Jiancheng Bioengineering Institute	/	/
Triglyceride (TG) assay kit	20231113	/	Nanjing Jiancheng Bioengineering Institute	/	/
Malondialdehyde (MDA) assay kit	20240229	/	Nanjing Jiancheng Bioengineering Institute	/	/

superoxide Dismutase (SOD) assay kit	202402	/	Nanjing Jiancheng Bioengineering Institute	/	/
Leptin assay kit	EE0012	/	Jingmei Biotechnology Co., Ltd.	/	/
Adiponectin assay kit	/	/	Nanjing Jiancheng Bioengineering Institute	/	/
Hematoxylin–eosin stain kit	HFVTU	/	Jingmei Biotechnology Co., Ltd.	/	/
RNA tissue/cell rapid extraction kit	HTBKZ	/	Shandong SparkJade Biotechnology Co., Ltd.	/	/
SPARKscript II all-in-one RT SuperMix for qPCR（with gDNA eraser）	HUKNU	/	Sparkjade Science Co., Ltd., China	/	/
2×SYBR Green qPCR Mix（with ROX）	/	/	Sparkjade Science Co., Ltd., China	/	/

[a] High-performance liquid chromatography. [b] Gas chromatography.

Table S7 Formula for high fat feed

Formula	Weight percent (%)
Maintain rat feed powder	49.3
Sucrose	20.0
Lard	15.0
Casein（85%）	12.5
Cholesterol	1.2
Calcium hydrogen phosphate	1.3
Rock powder	0.5
Sodium cholate	0.2

Table S8 Groups and dosages

Group	Abbreviation	Dosage (mg/kg)
Normal control group	NCG	
High fat diet group	HFD	
Positive control group (*L*(-)-Carnitine)	L-C	100
HYP-MAL low dose group	HYP-MAL-L	37.06
HYP-MAL medium dose group	HYP-MAL-M	74.12
HYP-MAL high dose group	HYP-MAL-H	111.18

Table S9 Detection of primer sequence information

Name	Sequence (5'→3')
LEP-F	CCTGCCTTCCCAAAATGTGC
LEP-R	TTGGAGAAGGCCAGCAGATG
ADP-F	TGTTCCTCTTAATCCTGCCCA
ADP-R	CCAACCTGCACAAGTTCCCTT
GAPDH-F	GCTGGAGTCTTGTCAGGCAT
GAPDH-R	CCCTGTACATGCTGGAGTTGAG

NMR and elemental analysis data of HYP-MAL: ^{1}H NMR (400 MHz, DMSO-d6) δ 8.15 (s, 1H), 7.98 (s, 1H), 6.26 (s, 2H).

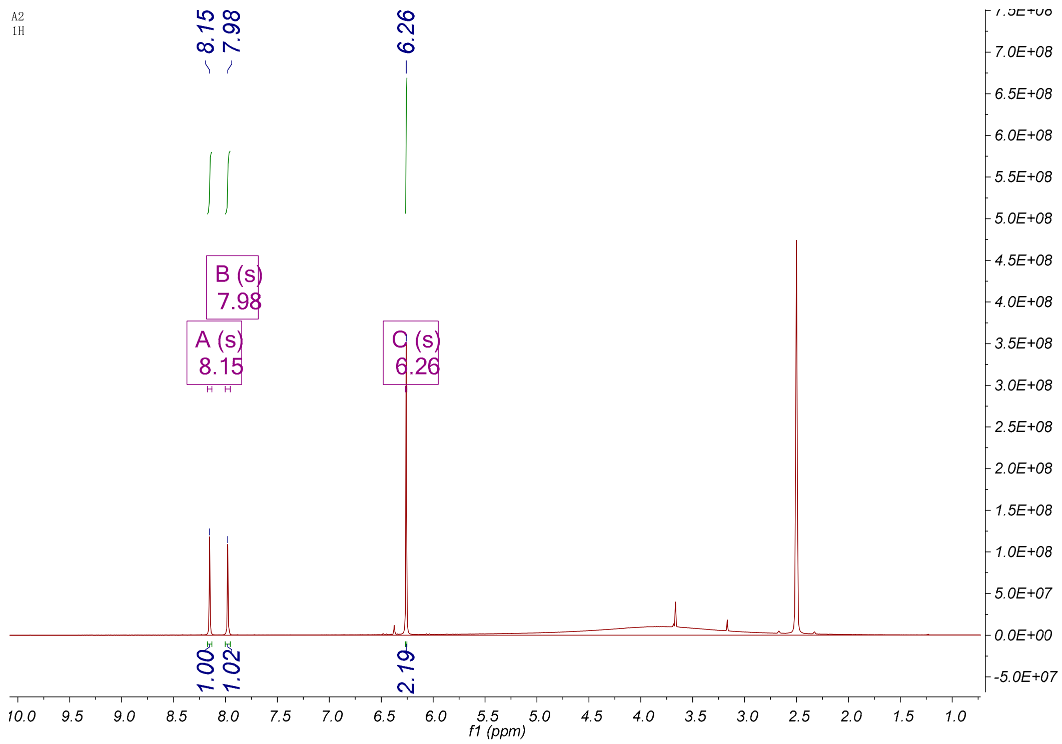

Figure S1. ^{1}H NMR spectra of HYP-MAL.

NMR and elemental analysis data of HYP: ^{1}H NMR (400 MHz, DMSO-d6) δ 8.11 (s, 1H), 7.97 (s, 1H).

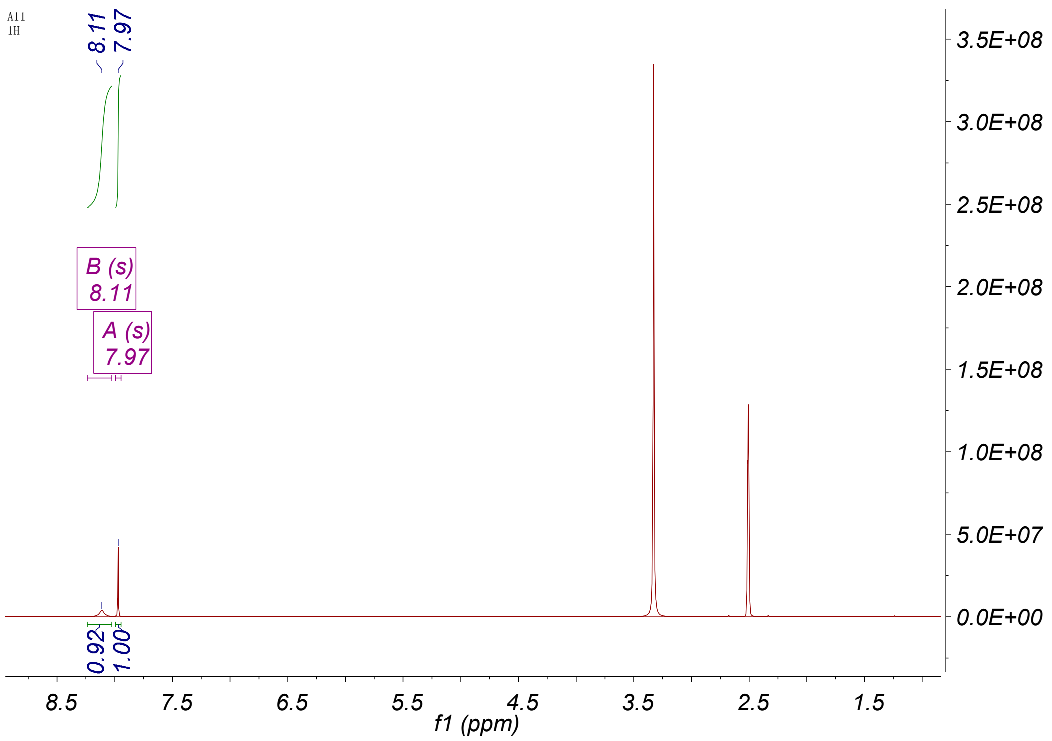

Figure S2. ^{1}H NMR spectra of HYP.

NMR and elemental analysis data of MAL: ^{1}H NMR (400 MHz, DMSO-d6) δ 6.27 (s, 1H).

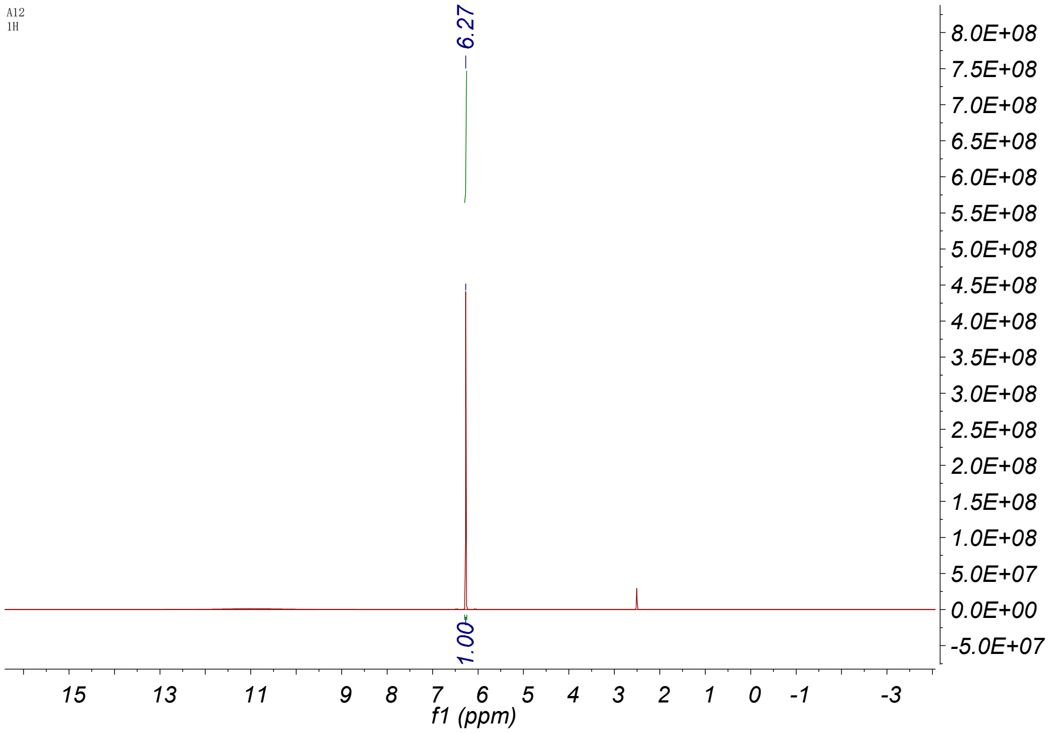

Figure S3. ^{1}H NMR spectra of maleic acid.

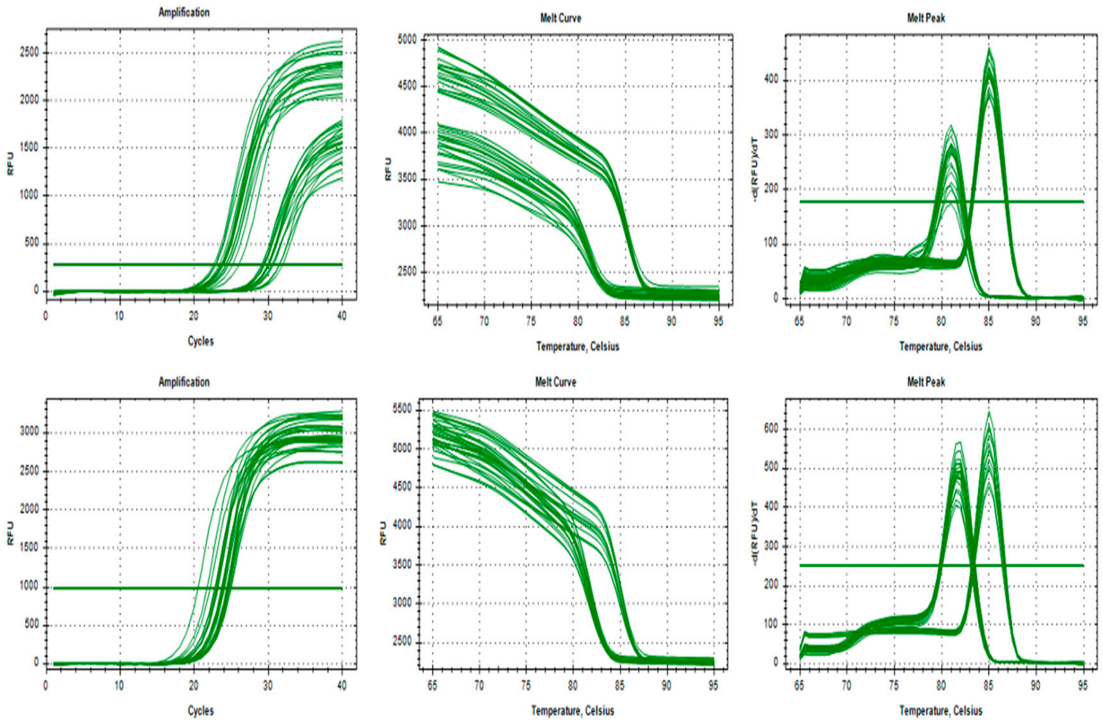

Figure S4. Amplification and dissolution curves of (a) LEP and (b) ADP
